# Divergence of Liver Lipidomes in Tibetan and Yorkshire Pigs Living at Different Altitudes

**DOI:** 10.3390/molecules28072991

**Published:** 2023-03-27

**Authors:** Wei Luo, Yisha Xu, Xuedong Gu, Jiamin Zhang, Jinqiu Wang, Fang Geng

**Affiliations:** 1 Meat Processing Key Laboratory of Sichuan Province, School of Food and Biological Engineering, Chengdu University, Chengdu 610106, China; 2 College of Food Science, Tibet Agriculture and Animal Husbandry University, Linzhi 860000, China

**Keywords:** Tibetan pigs, Yorkshire pigs, liver, lipidome, triglycerides, phospholipids

## Abstract

The Tibetan pig is a characteristic breed of the Qinghai-Tibet Plateau with distinct physiological and meat quality attributes. The liver lipid profile can offer an important perspective to explore the uniqueness of Tibetan pigs. A quantitative comparison of liver lipidomes revealed significant differences in the lipid profiles between Tibetan and Yorkshire pigs raised at different altitudes. The abundance of lipids in the livers of pigs raised at a high altitude was higher than that of pigs raised at a lower altitude, whereas the abundance of lipids in the livers of Yorkshire pigs was higher than that of Tibetan pigs raised at the same altitude. Of the 1101 lipids identified, 323 and 193 differentially abundant lipids (DALs) were identified in the pairwise comparisons of Tibetan and Yorkshire pigs raised at different altitudes, respectively. The DALs of Tibetan pigs consisted mainly of 161 triglycerides, along with several acylcarnitines, represented by carnitine C2:0, and significant changes in the abundance of some phospholipids. The DALs of Yorkshire pigs were more complex, with significant increases in the abundance of triglycerides, cholesteryl esters, and free fatty acids, and decreases in the abundance of some phospholipids. This research provides strong theoretical and data support for the high-quality development of the highland livestock industry.

## 1. Introduction

The Tibetan pig breed is mainly raised on the Qinghai-Tibetan plateau in China [1]. Due to their long-term exposure to an alpine and anoxic environment, Tibetan pigs show different characteristics and advantages from commercial pigs bred in other low-altitude areas in China in terms of living habits, body organization, and physiological regulation [2]. Accordingly, the meat attributes, including the taste and flavour, of Tibetan pigs also show great differences from those of other pig breeds. For example, studies have found significant differences in the volatile compound fractions of boiled pork from Tibetan, Sanmenxia, and Duroc × (local × Yorkshire) pigs [3]. Furthermore, the edible qualities of Tibetan pork (such as tenderness, juiciness, overall preference, and cooking loss) are significantly associated with its whole-genome sequence and single nucleotide polymorphisms [4]. Wang and colleagues [5] compared the protein profiles between Tibetan and Yorkshire pig loin and found the differential expression of several proteins in the Tibetan pig loin associated with pathways of energy production, muscle contraction, immunity and defence, and glutathione metabolism, which are highly correlated with meat signature formation. The development of the unique meat attributes of Tibetan pigs, therefore, appears to be closely linked to their adaptive regulation in high-altitude environments [6]. However, the molecular mechanisms underlying this adaptive regulation remain elusive.

Several in-depth studies have been conducted to further understand the regulatory control mechanisms of adaptation to the plateau environment in Tibetan pigs. These investigations have revealed the important roles of various tissues and organs of the Tibetan pig in the survival process during adaptation to the highland environment. For example, the heart of the Tibetan pig can be physiologically regulated at several levels (e.g., increased expression of erythropoietin and endothelial PAS structural domain-containing protein 1 (EPAS1)) to ensure blood circulation under extreme hypoxic conditions [7]. The genes *KLF4*, *BCL6B*, *EGR1*, *EPAS1*, *SMAD6*, *SMAD7*, *KDR*, *ATOH8*, and *CCN1* have also been identified as potential regulators of hypoxia adaptation in the Tibetan pig lung tissue [8]. Moreover, Tibetan pigs exhibit greater cardiac and hepatic resistance to oxidative stress than Yorkshire pigs by actively regulating the AMPK/p38 MAPK/Nrf2-ARE signalling pathway under hypoxic conditions on the plateau [9].

As a major organ that regulates energy supply and performs physiological functions such as detoxification, the liver’s internal physiological regulatory processes are closely linked to changes in the external environment [10]. A previous transcriptomics and proteomics comparative analysis of the livers of Tibetan and Yorkshire pigs found significant differences in small-molecule biosynthesis, metabolic processes, and the metabolism of organic hydroxyl compounds [11]. In addition, as the centre of lipid metabolism, the liver plays a very important role in the regulation of lipid-related substances in living organisms [12]. In contrast to the lipid deposition function performed primarily by the adipose tissue [13], the liver is also involved in the catabolism of lipids, which is important for maintaining an efficient supply of energy and facilitating the adaptation of the body to thermogenesis. Li and colleagues [14] used transcriptomics to compare the differences in lipid metabolism in the livers of Tibetan and lake sheep, showing that Tibetan sheep have higher levels of fatty acid (FA) oxidation in the liver. Changes in the expression of some key genes further suggested that hepatic lipid metabolism plays an important role in adaptive thermogenesis in Tibetan sheep [14]. In addition to being an important source of energy storage, lipids have other key biological roles such as being structural components of the plasma membrane and as intermediates in metabolic and signalling pathways [15]. A comparison of characteristic lipid differences in liver tissues will therefore provide further information to reveal the molecular mechanisms underlying phenotypic differences. Mi and colleagues [16] tentatively identified significant differences in the lipid composition of Tibetan, Jilin, and Sanmenxia pigs. However, elucidating the mechanisms driving these observed differences requires further investigation.

Finally, as a regional speciality with a high nutritional value, Tibetan pork has been increasingly sought after by consumers [5,17]. However, the complex ecological environment in the highlands remains an important limiting factor for pig production [9]. Therefore, further exploration of the physiological mechanisms of adaptation of Tibetan and Yorkshire pigs to the plateau environment will not only help to understand the impact of the changes in the ecological environment of the plateau on the survival of these breeds but can also provide a valuable reference for local pork food processing and genetic improvement of native pigs to, in turn, promote the further development of the livestock industry with plateau characteristics.

In this study, the ultra-high-performance liquid chromatography tandem mass spectrometry (UPLC-MS/MS) technique was used to quantitatively compare the liver lipidomes of Tibetan and Yorkshire pigs raised at different altitudes to further elucidate the adaptive molecular regulatory mechanisms underlying the differences in the adaptive capacity of the two breeds to the highland environment. These data can provide theoretical guidance for the subsequent high-quality development of the highland livestock industry.

## 2. Results and Discussion

### 2.1. Liver Lipidomic Identification of Pig Breeds at Different Altitudes

**Figure 1:**
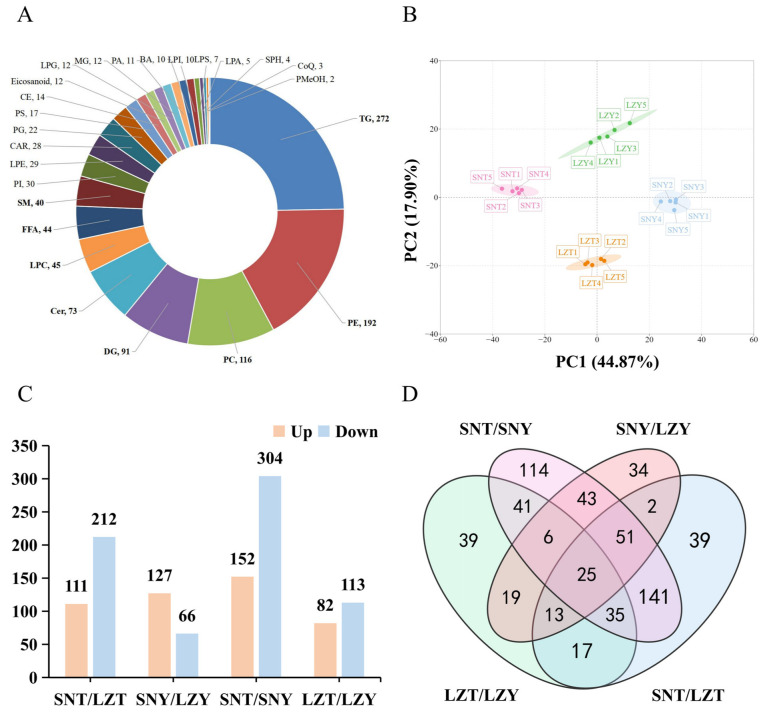
Lipidomic analysis of the pig liver of different breeds raised at different altitudes. (**A**) Number of lipids for the 25 lipid subclasses identified in the LZT, SNT, LZY, and SNY groups. (**B**) Principal component analysis (PCA). (**C**) The number of differentially abundant lipids (DALs) obtained by comparisons between two groups. (**D**) Venn diagram of the DALs distribution.

Firstly, the results of the quality control analysis of the mixed samples showed that the experimental instrumentation was very stable, providing important assurance of the reproducibility and reliability of the data for subsequent lipidomics analysis (Figure S1 and S2, Supplementary Materials). A total of 1101 lipids were detected in the four groups (LZT, SNT, LZY, and SNY), belonging to six major lipid classes (sterolipids, fatty acyl esters, sphingolipids, isopentenolipids, glycerolipids, and glycerophospholipids) and 25 lipid subclasses, including 272 triglycerides (TGs), 192 phosphatidylethanolamines (PEs), 116 phosphatidylcholines (PCs), 91 diglycerides (DGs), 73 ceramides (Cers), 45 lysophosphatidylcholines (LPCs), 44 free fatty acids (FFAs), 40 sphingomyelins (SMs), 30 phosphatidylinositols (PIs), 29 lysophosphatidylethanolamines (LPEs), and 28 acylcarnitines (CARs) (Figure 1A). Similarly, Mi et al. [16] identified 1180 lipids by liquid chromatography-MS/MS to characterise the lipid profiles of Tibetan, Jilin, and Sanmenxia black pig pork cuts (shoulder, rump, loin, shank, and belly).

### 2.2. Multivariate Statistical Analysis of the Pig Liver Lipidome According to Altitude and Breed

First, multivariate analysis was used to assess the overall differences in the liver lipidomes from different breeds of pigs grown at different altitudes [18]. The overall principal component analysis (PCA) results showed that all five replicates of each group of samples formed clusters (Figure 1B), indicating the reliability of the lipidomic analysis. The four sample groups showed clear separation, with 44.87% and 17.90% of the total variance explained by principal components 1 and 2, respectively. The grouped PCA analysis comparing the four groups of samples two by two further highlighted the between-group differences (Figure S3A–D, Supplementary Materials). These results indicated that altitude has a significant effect on the liver lipid profile of both Tibetan and Yorkshire pigs along with significant differences in the liver lipidomes of the two breeds raised at the same altitude.

The lipidomic data of the four groups of samples were further analysed by orthogonal partial least-squares discrimination analysis (OPLS-DA) [19,20]. The OPLS-DA score maps for LZT and SNT liver lipidomes were clearly differentiated (Figure S3E) and the OPLS-DA model had a predictive value of 98.2% (Q^2^ = 0.982) for distinguishing Tibetan pig liver lipidomes from different altitudes. Similarly, the OPLS-DA model revealed a 67.5% (R^2^X = 0.675) difference in the lipidomic of Yorkshire pig livers (LZY and SNY) at different altitudes (Figure S3F; R^2^Y = 0.998, Q^2^ = 0.970). Moreover, the OPLS-DA analysis showed greater differences in the liver lipidomes between Tibetan and Yorkshire pigs at high altitudes than at low altitudes (Figure S3G–H).

### 2.3. Changes in the Abundance of Major Lipid Classes in the Pig Liver According to Altitude and Breed

**Figure 2:**
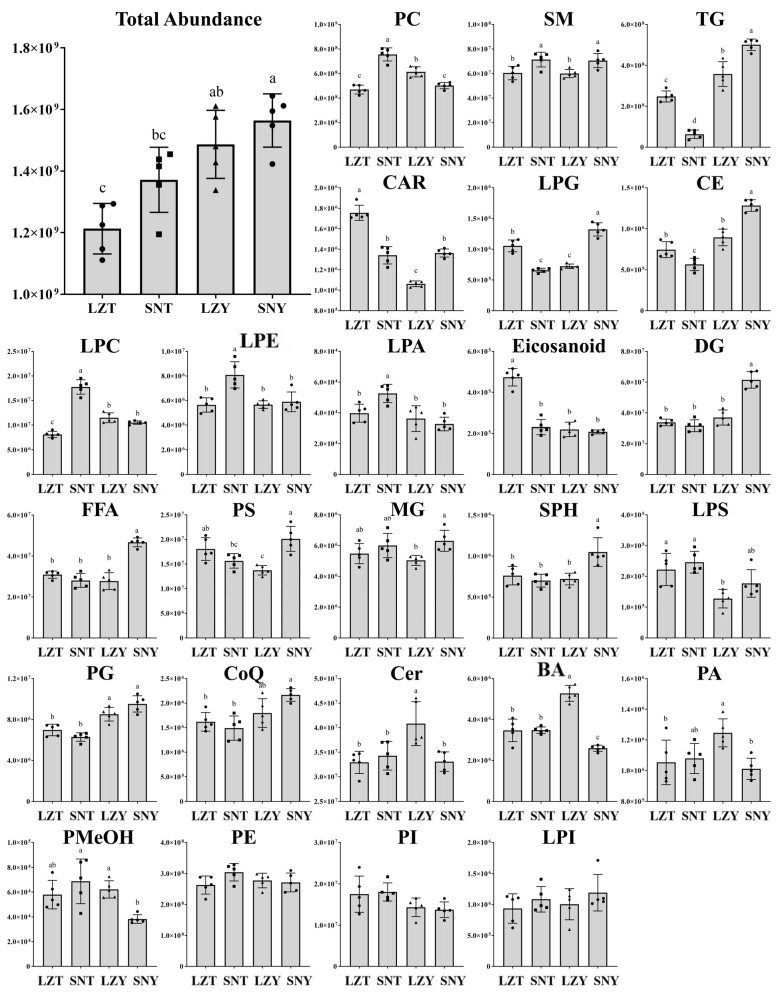
Comparison of the total lipid abundance and abundances of 25 lipid subclasses in the pig liver of different breeds raised at different altitudes. The ordinate is the ion intensity of lipid molecules in the mass spectrum (count per second). Significant differences (*p* < 0.05) between the four groups are indicated by different lowercase letters (a–c).

The differences in total lipid abundance and the abundance of each lipid subclass in the livers of different pig breeds grown at different altitudes were further compared (Figure 2). There were some differences in total liver lipid abundance between pigs of the same breed grown at different altitudes, although they were not significant (*p* > 0.05), whereas there were significant differences in the liver lipid abundance between pigs of different breeds grown at the same altitude (*p* < 0.05), with Yorkshire pigs all having a higher liver lipid abundance than Tibetan pigs. The differences in liver lipid metabolism may also reflect, to some extent, differences in the feed conversion and growth rates between breeds of pigs. In particular, Yorkshire pigs show a higher feed conversion rate and growth rate (shorter slaughter time, 3–4 months) compared to Tibetan pigs (longer slaughter time, 10–12 months) [21]. In addition, the greater reserve of lipid fractions in the liver of Yorkshire pigs may facilitate their initial adaptation to high-altitude cold environments, but further investigation is required.

The abundance of various subclasses of lipids in the liver of pigs of the same breed grown in different altitudinal environments also varied greatly (Figure 2). For example, the abundances of PCs, SMs, LPCs, LPEs, and LPAs in the livers of Tibetan pigs grown at high altitude (SNT) were significantly higher than those of Tibetan pigs grown at low altitude (LZT, *p* < 0.05), whereas the abundances of TGs, CARs, lysophosphatidyl glycerol (LPG), cholesteryl esters (CEs), and eicosanoid in SNT were significantly lower than those of LZT. In addition, the abundances of SMs, TGs, CARs, LPGs, CEs, DGs, FFAs, phosphatidylserines, monoglycerides, and sphingosines in pig livers from Yorkshire pigs grown at high altitude (SNY) were significantly higher than those in low-altitude Yorkshire pigs (LZY, *p* < 0.05), whereas the abundance of PC, Cer, bile acid, phosphatidic acid, and phosphatidyl methanol in SNY were significantly lower than those of LZY. Further comparisons revealed distinct lipid profiles of Tibetan and Yorkshire pigs’ livers raised at different altitudes, with only SM showing a higher abundance in both breeds at higher than at lower altitudes, whereas PC, TG, CAR, LPG, and CE showed opposite patterns. Moreover, the abundance of more lipid classes in the livers of Yorkshire pigs changed significantly at different altitudes, which also potentially reflects the more extensive effects of altitude on their liver lipids and related physiological metabolism, and laterally indicates that this breed is less able to adapt to changes in altitude than Tibetan pigs.

### 2.4. Differentially Abundant Lipids (DALs) in the Pig Liver According to Altitude and Breed

#### 2.4.1. DALs in the Liver of Pigs of the Same Breed Raised at Different Altitudes

A total of 323 (111 upregulated, 212 downregulated) DALs (Figure 1C) were identified in the SNT/LZT comparison, belonging to 19 lipid subclasses. These mainly included 161 TGs (161 downregulated), 33 PCs (32 upregulated, 1 downregulated), 28 PEs (27 upregulated, 1 downregulated), 22 LPCs (19 upregulated, 3 downregulated), 14 CARs (1 upregulated, 13 downregulated), 12 DGs (6 upregulated, 6 downregulated), and 10 Cers (3 upregulated, 7 downregulated).

The 193 (127 upregulated, 66 downregulated) DALs (Figure 1C) found in the SNY/LZY comparison belong to each of the 21 lipid subclasses. These mainly included 25 PEs (9 upregulated, 16 downregulated), 22 TGs (20 upregulated, 2 downregulated), 21 PCs (7 upregulated, 14 downregulated), 17 CARs (11 upregulated, 6 downregulated), 15 DGs (14 upregulated, 1 downregulated), 14 LPCs (6 upregulated, 8 downregulated), 13 Cers (2 upregulated, 11 downregulated), 11 PIs (6 upregulated, 5 downregulated), and 10 FFAs (10 upregulated).

Although more DALs were present in the livers of Tibetan pigs grown at different altitudes (SNT/LZT) compared to those of Yorkshire pigs, they mainly belonged to the TG class of lipids, suggesting that TG metabolism was dramatically regulated in response to changes in altitude. However, further research and exploration are still needed. One study showed that acute exposure to reduced oxygen inhibited plasma TG clearance in animals, resulting in a significant increase in plasma TG [22]. In contrast, more lipid classes in the livers of Yorkshire pigs raised at different altitudes (SNY/LZY) showed significant changes, again suggesting, to some extent, that altitude changes have a broader effect on Yorkshire pig liver lipids and their associated physiological metabolism.

#### 2.4.2. DALs in the Liver of Different Pig Breeds Raised at the Same Altitude

A total of 456 (152 upregulated, 304 downregulated) DALs were found in the SNT/SNY comparison (Figure 1C). They belonged to 22 lipid subclasses, mainly consisting of 187 of TGs (187 downregulated), 49 PEs (42 upregulated, 7 downregulated), 41 PCs (29 upregulated, 12 downregulated), 35 DGs (8 upregulated, 27 downregulated), 25 LPCs (15 upregulated, 10 downregulated), 17 Cers (7 upregulated, 10 downregulated), 15 PIs (12 upregulated, 3 downregulated), 13 LPEs (11 upregulated, 2 downregulated), 10 CARs (5 upregulated, 5 downregulated), and 10 PGs (3 upregulated, 7 downregulated).

A total of 195 (82 upregulated, 113 downregulated) DALs were identified in the LZT/LZY comparison (Figure 1C), belonging to 18 lipid subclasses, including mainly 67 TGs (13 upregulated, 54 downregulated), 18 PEs (8 upregulated, 10 downregulated), 17 CARs (15 upregulated, 2 downregulated), 17 DGs (7 upregulated, 10 downregulated), 16 PCs (1 upregulated, 15 downregulated), and 13 LPCs (2 upregulated, 11 downregulated).

Overall, this comparison revealed more significant differences in liver lipids between Tibetan and Yorkshire pigs at higher altitudes compared to lower altitudes. This may be due to the fact that at high altitudes the two breeds of pigs show greater differences in adapting to the drastic changes in the external environment. Some differences in gene regulation during adaptation to environmental change have been tentatively identified in previous studies between these two breeds of pigs, particularly in high-altitude environments [23].

### 2.5. Kyoto Encyclopaedia of Genes and Genomes (KEGG) Annotation and Enrichment Analysis of Pig Liver DALs According to Altitude and Breed

**Figure 3:**
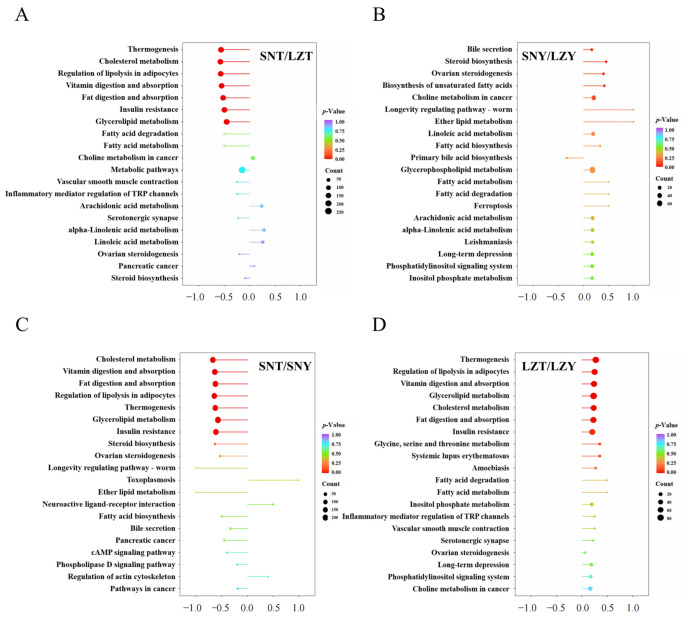
KEGG analysis of pig liver DALs for different breeds raised at different altitudes. (**A**) SNT/LZT; (**B**) SNY/LZY; (**C**) SNT/SNY; and (**D**) LZT/LZY. The x-axis represents the differential score, the differential abundance score = (number of differential metabolites upregulated in the pathway—number of differential metabolites downregulated in the pathway)/number of all metabolites annotated to the pathway.

To further understand the physiological processes involved and the functions performed by porcine liver DALs, they were subjected to KEGG annotation and enrichment analysis. The 323 DALs in the SNT/LZT comparison were annotated to 46 KEGG pathways, 7 of which were significantly enriched (*p* < 0.01), including ‘Thermogenesis’, ‘Cholesterol metabolism’, ‘Regulation of lipolysis in adipocytes’, ‘Vitamin digestion and absorption’, ‘Fat digestion and absorption’, ‘Insulin resistance’, and ‘Glycerolipid metabolism’ (Figure 3A). The 193 DALs in the SNY/LZY comparison were annotated to 57 KEGG pathways, 4 of which (‘Bile secretion’, ‘Steroid biosynthesis’, ‘Ovarian steroidogenesis’, and ‘Biosynthesis of unsaturated fatty acids’) were significantly enriched (*p* < 0.05; Figure 3B).

In addition, the 456 DALs identified in the SNT/SNY comparison were annotated to 107 KEGG pathways, with 7 pathways significantly enriched (*p* < 0.01), including ‘Cholesterol metabolism’, ‘Vitamin digestion and absorption’, ‘Fat digestion and absorption’, ‘Regulation of lipolysis in adipocytes’, ‘Thermogenesis’, ‘Glycerolipid metabolism’, and ‘Insulin resistance’ (Figure 3C). In contrast, the 195 DALs found in the LZT/LZY comparison were only annotated to 41 KEGG pathways, and 7 pathways, including ‘Thermogenesis’, were significantly enriched (*p* < 0.05; Figure 3D).

Further comparison revealed that the DALs in the livers of both Tibetan pigs grown at different altitudes (SNT/LZT) and of Tibetan and Yorkshire pigs raised at the same altitude (SNTSNY and LZT/LZY) were significantly enriched in ‘Thermogenesis’, ‘Cholesterol metabolism’, ‘Regulation of lipolysis in adipocytes’, ‘Vitamin digestion and absorption’, ‘Fat digestion and absorption’, ‘Insulin resistance’, and ‘Glycerolipid metabolism’. In contrast, the DALs in the livers of Yorkshire pigs raised at different altitudes SNY/LZY) were only significantly enriched in four KEEGG pathways, including ‘Bile secretion’, ‘Steroid biosynthesis’, ‘Ovarian steroidogenesis’, and ‘Biosynthesis of unsaturated fatty acids’, showing significant differences from the pattern found in Tibetan pigs. Metabolic pathways such as ‘Thermogenesis’ and ‘Glycerolipid metabolism’ have been found to play an important role in the adaptation of animals to changes in altitude [24,25,26].

### 2.6. Complex Lipid Metabolism in the Liver of Tibetan and Yorkshire Pigs Reflects Adaptation to Environmental Changes

Differences in the genetic background of Tibetan and Yorkshire pigs lead to a different performance in their adaptation to the highland environment, and the phenotype of liver lipid metabolism in animals may reflect the regulatory mechanisms behind the phenotypic differences to some extent [14]. Therefore, we further compared the liver lipid composition of Tibetan and Yorkshire pigs raised at different altitudes to provide additional insight into the differences in the molecular mechanisms of lipid metabolism between these two breeds in adapting to the cold environment of the plateau.

#### 2.6.1. Efficient Lipid Catabolism Provides Energy Support for the Adaptive Survival of Tibetan Pigs in Cold Environments

**Figure 4:**
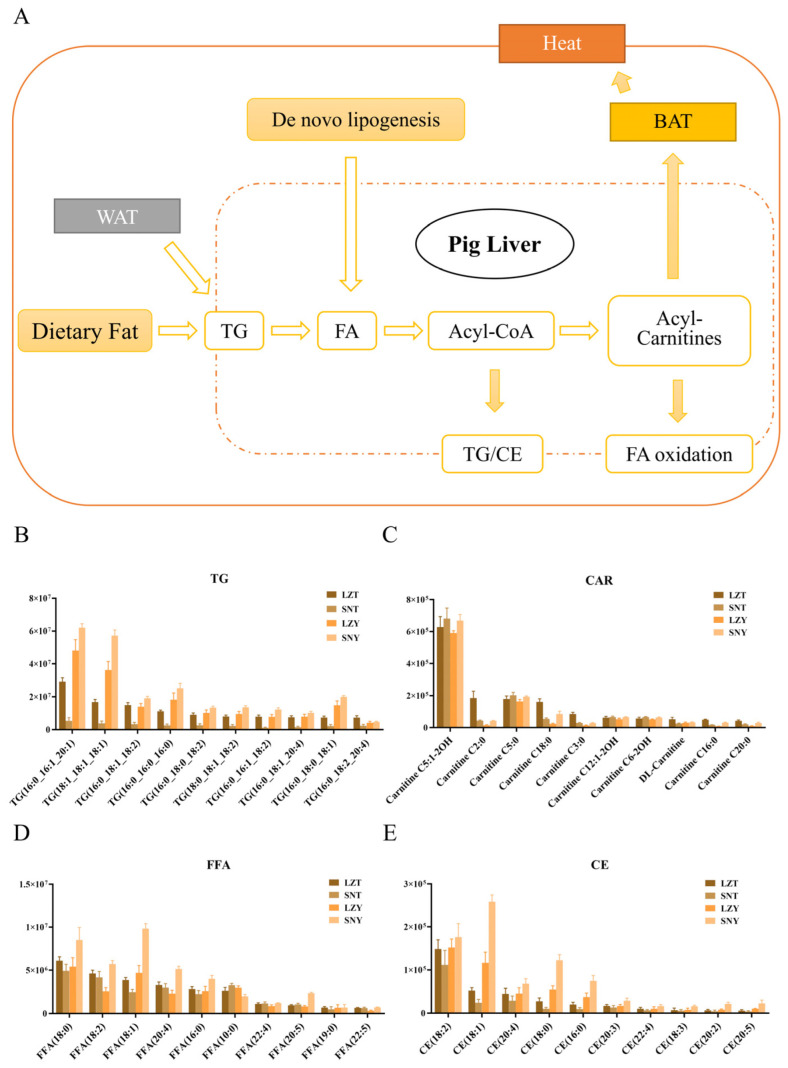
Complex lipid metabolism in the liver of Tibetan and Yorkshire pigs. (**A**) Schematic diagram of the lipid metabolism processes occurring in the pig liver. (**B**) Top 10 abundant triglycerides (TGs) in the pig liver. (**C**) Top 10 abundant carnitines (CARs) in the pig liver. (**D**) Top 10 abundant free fatty acids (FFAs) in the pig liver. (**E**) Top 10 abundant cholesteryl esters (CEs) in the pig liver.

As an important energy substance, lipids play an essential role in the normal metabolism of the body and metabolic regulation in response to adverse environments, and the liver is the central hub of lipid metabolism [11,14]. Some important physiological processes such as uptake, esterification, oxidation, and secretion of fatty FAs all occur in liver cells [12]. Based on existing research findings, the lipid metabolism process that takes place in the Tibetan pig liver can be broadly divided into the following steps (Figure 4A).

In the first step, lipids such as TG, which originate from dietary intake, adipose tissue breakdown, and de novo synthesis, are broken down into FAs [14]. In the present study, the total TG abundance in SNT was significantly lower than that in LZT (Figure 2), suggesting that Tibetan pigs have less TG reserves in the liver during growth at higher altitudes compared to lower altitudes, which may also be due to more TG molecules entering the subsequent lipid metabolism process. Furthermore, 161 of the 272 TGs identified showed a significantly different abundance in SNT and LZT, and all of them showed a decrease in abundance, including the top 10 TGs (Figure 4B).

In the second step, FAs obtained from lipolysis such as TG are activated to acyl-coenzyme A, which then proceeds to the next step in the metabolic process, mainly consisting of beta-oxidation or complex lipid biosynthesis [27]. Although there was no significant difference in the total FFA abundance between SNT and LZT, four long-chain FFAs, FFA (29:0), FFA (30:0), FFA (36:0), and FFA (38:1), showed significantly reduced abundance in SNT vs. LZT (Figure 2). The top 10 FFAs were also significantly less abundant in SNT (18:1) than in LZT (Figure 4D). Combined with the decreasing trend in TG, more FAs in the liver of Tibetan pigs raised at a high altitude are activated to the next process in lipid metabolism. Furthermore, since the FA activation reaction is catalysed by members of the long-chain acyl coenzyme A synthase (ACSL) family of enzymes, the expression of different acyl coenzyme A synthases may affect the course of activated FAs [28]. For example, the upregulation of ACSL4 expression and the downregulation of ACSL1 and ACSL5 expression in the liver of Tibetan sheep compared to those of lake sheep seem to indicate that FA activation in the liver of Tibetan sheep is more focused on lipid oxidation processes than on lipid deposition [14]. Zhu et al. [29] also found the downregulated expression of genes controlling lipid deposition in the muscles of Tibetan pigs, whereas genes mainly involved in lipid metabolism and skeletal muscle growth were up-regulated compared to those of common domestic pigs at lower altitudes.

In the third step, acyl-coenzyme A is converted to acyl-carnitine, which can be further broken down by the brown adipose tissue (BAT) to produce heat [30,31]. In this study, the total abundance of CARs was significantly reduced in SNT compared to LZT, with 13 of the 28 CARs identified having reduced abundance (Figure 2). Many of the top 10 CARs also showed a decrease in abundance, including carnitine C2:0, which is directly derived from acetyl coenzyme A and decreased in abundance by nearly 5-fold (Figure 4C). This suggests that in response to the cold environment, more CARs in the liver of Tibetan pigs at high altitudes are provided to the BAT to generate heat. It has been reported that cold environmental conditions induce an overall change in body lipid metabolism, thereby increasing the transport of FAs to the BAT through various pathways, with indirect internalization in the BAT after conversion from the liver to acylcarnitine, representing an important pathway for lipid thermogenesis [32].

In summary, this study shows differences in the lipid metabolism in Tibetan pigs raised at different altitudes, with those raised at higher altitudes undergoing more efficient FA oxidation to actively produce energy to resist cold conditions and maintain body temperature. This is another indication that Tibetan pigs have evolved a more efficient lipid metabolism system for environmental adaptation that is economical and practical, which is important for adaptation to the harsh environment of extreme cold and lack of food sources on the plateau.

#### 2.6.2. Efficient Lipid Conversion and Accumulation Provide the Material Basis for the Adaptive Survival of Yorkshire Pigs in Cold Environments

In contrast to the adaptation of Tibetan pigs to environmental changes by engaging in more efficient lipolysis metabolism, Yorkshire pigs have a distinct regulation mechanism for living at different altitudes.

Firstly, the total TG abundance was significantly higher in SNY than in LZY (Figure 2), which was the opposite trend found for Tibetan pigs, and the total TG abundance in Yorkshire pig livers was significantly higher than that of Tibetan pig livers in all cases. Of the 272 TGs identified, only 22 showed significantly different abundance in SNY/LZY, with 20 increasing and 2 decreasing, and the top 10 TGs all showed an increasing trend in abundance (Figure 4B).

The activation of FAs to acyl-coenzyme A generally follows a two-way process: on the one hand, FAs in the liver can be converted to TG and CE, subsequently secreted as, for example, very-low-density lipoprotein particles; on the other hand, they can be broken down step-by-step through the process of lipid oxidation and release energy to enable the body’s physiological processes to proceed efficiently [33,34]. In this study, the total FFA abundance was significantly higher in SNY than in LZY (Figure 2). Correspondingly, a significant increase in the abundance of 10 FFAs was found in the SNY/LZY comparison. As a result, the livers of Yorkshire pigs raised at a high altitude produce more FAs to cope with the high intensity of lipid metabolism in cold environments than found for the more ‘compact’ Tibetan pigs. Further comparison of the changes in the abundance of TG and CE (Figure 2 and Figure 4B, E) indicated that the process of lipid synthesis towards TG/CE is similarly further enhanced in the liver of Yorkshire pigs at high altitudes compared to that of high-altitude Tibetan pigs, where the conversion to TG/CE is weakened and thus more favoured towards FA oxidation. This difference further explains the higher total lipid abundance in the liver of Yorkshire pigs compared to that of Tibetan pigs.

In addition to the significantly higher abundance of CARs in SNY than in LZY, 11 of the 28 CARs in SNY/LZY showed a significant increase in abundance, with the top 10 CARs showing the same trend (Figure 2 and Figure 4C). This shows that although Yorkshire pigs at a high altitude intensify their lipid deposition, they also spontaneously produce more CARs for subsequent heat production and supply in response to the cold environment at high altitudes due to the accumulation of the previous base.

In summary, this study further demonstrates differences in the mechanism by which the liver lipids of different pig breeds are regulated in response to changes in the external environment. Specifically, Tibetan pigs are more inclined to undergo FA oxidation than adipogenesis in lipid metabolism. Therefore, Tibetan pigs are likely to be more efficient than Yorkshire pigs in terms of the conversion process of lipid metabolism to energy. However, in terms of lipid accumulation efficiency, Yorkshire pigs are significantly more efficient than Tibetan pigs. Moreover, Yorkshire pigs raised in high-altitude environments not only undergo more lipid deposition but also spontaneous lipid oxidation to maintain body temperature. This may be due to the fact that the metabolic system of substances in the body of long-selected Yorkshire pigs, which is adapted to a low-altitude environment and the demands of meat production, is more focused on the synthesis and accumulation of substances. Even when raised in the highland environment, Yorkshire pigs do not exhibit the same efficient environmental adaptive regulation as Tibetan pigs due to constraints from their genetic and molecular basis and can only achieve environmental adaptive regulation by further enhancing their metabolic level. However, this is undoubtedly a crude regulation mechanism that requires support from enhanced digestion and the absorption of more food.

### 2.7. Potential Effects of Adaptive Regulation of Liver Lipid Metabolism on Dietary Nutritional Value and Meat Quality Attributes

**Figure 5:**
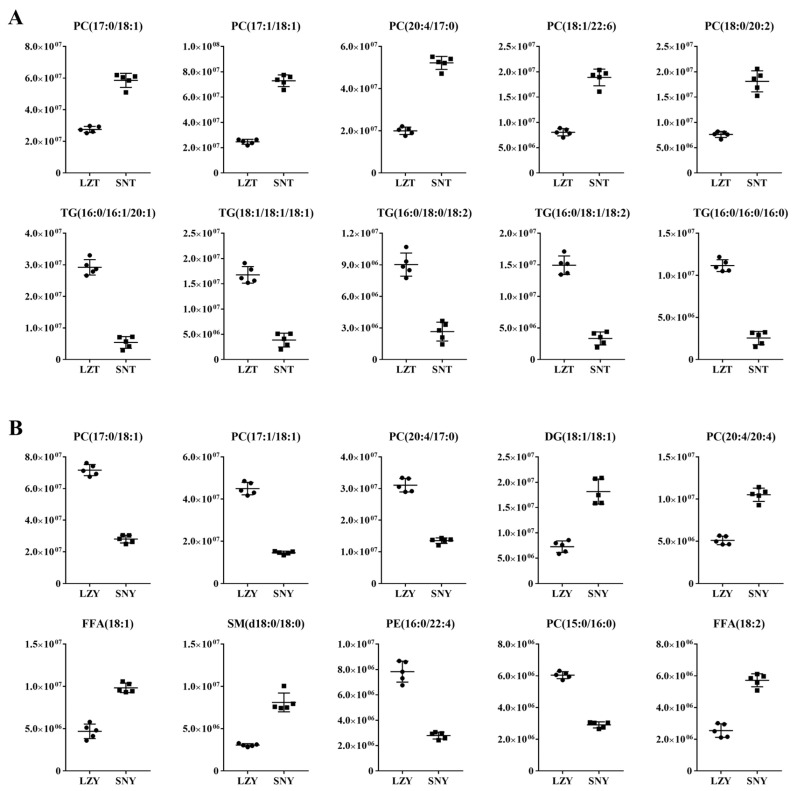
Representative DALs in the livers of Tibetan and Yorkshire pigs raised at different altitudes. (**A**) SNT/LZT and (**B**) SNY/LZY.

Further comparative analysis of the DALs of SNT/LZT revealed that the top 10 lipids in abundance included 5 phospholipids and 5 TGs (Figure 5A). As mentioned above, TG in the Tibetan pig liver plays an important role in lipid metabolism and energy supply. The significant increase in the abundance of five representative phospholipids in the livers of high-altitude Tibetan pigs, such as PC (17:0_18:1), PC (17:1_18:1), PC (20:4_17:0), PC (18:1_22:6), PC (18:0_20:2), and especially the polyunsaturated fatty acids (PUFAs) PC (20:4_17:0) and PC (18:1_22:6), may have contributed to the improvement of the anti-oxidative stress regulation capacity of the liver on the one hand [9], while further improving the nutritional value of high-altitude Tibetan pig products on the other hand. This is because PUFAs have a variety of physiological functions such as maintaining biofilm structure, treating cardiovascular disease, having anti-inflammatory effects, and promoting brain development [35]. For highlanders, whose food sources are relatively homogeneous, a further increase in the PUFA/saturated fatty acid (SFA) ratio in food would be more beneficial to health [36]. The further enrichment of PUFAs in high-altitude Tibetan pigs thus effectively complements the nutritional needs of the local people’s diet and strongly reflects the long-term selection and harmonious evolution of humans and pigs.

For Yorkshire pigs raised at different altitudes, the typical DALs in their livers showed more complex changes (Figure 5B). Five of the phospholipids, PC (17:0_18:1), PC (17:1_18:1), PC (20:4_17:0), PE (16:0_22:4), and PC (15:0_16:0), showed significantly reduced abundance, whereas PC (20:4_20:4), DG (18:1_18:1), SM (d18:0/18:0), and two FAs [FFA (18:1) and FFA (18:2)] significantly increased in abundance in the livers of Yorkshire pigs raised at high altitude. These complex changes in the abundance of unsaturated phospholipid molecules reflect a relative reduction in the regulation of oxidative stress carried out by the liver on the one hand [9], and the unsuitability of Yorkshire pigs at high altitude as a long-term dietary option for people in highland areas on the other hand. Further enhancement of the lipid deposition in the livers of Yorkshire pigs at high altitudes along with a significant reduction in the number of some highly abundant unsaturated phospholipid molecules reflect the high capacity of Yorkshire pigs raised at low altitudes for lipid deposition and a high proportion of SFAs [6]. In contrast, existing studies have shown that the long-term consumption of meat products rich in lipids and SFAs can affect the normal metabolism of cholesterol in the body and cause a nutritional burden [37]. In addition, the intramuscular lipid content is closely related to meat quality traits such as tenderness, pH, marbling, and muscle flavour [38,39]. Therefore, changes in the lipid fraction in the liver of Yorkshire pigs raised at different altitudes also have a potential impact on the palatability quality of their liver or muscle tissue.

In conclusion, the results of this study show that the FA composition of Tibetan pig livers from high altitudes is more beneficial to the health of people on the plateau and that the livers of Yorkshire pigs raised at high altitudes are more enriched with more complex FAs, which can meet the dietary nutritional needs of specific populations to a certain extent. Similar findings were found in a previous study comparing meat quality traits and their FA composition in pig breeds living at three different altitudes (Yorkshire, Green Jade, and Tibetan) [6].

## 3. Materials and Methods

### 3.1. Chemicals and Reagents

Organic solvents (high-performance liquid chromatography (HPLC)-grade acetonitrile, methanol, isopropanol, tert-butyl methyl ether) for sample preparation and lipidome analysis were purchased from Merck Chemical Reagent Co., Ltd. (Shanghai, China). Formic acid (HPLC-grade) was purchased from Sigma-Aldrich (St. Louis, MO, USA). Ultrapure water was prepared using a Milli-Q system (Millipore, Billerica, MA, USA). Lipid standards (12:0 lysophosphatidylcholine, ceramide (d18:1/4:0), phosphatidylcholine (13:0/13:0), diglyceride (12:0/12:0), and triglyceride (17:0/17:0/17:0) were purchased from Avanti Polar Lipids (Alabaster, AL, USA).

### 3.2. Sample Collection

Tibetan pigs (12 months old, 30–35 kg) were raised in Shannan (SNT; altitude of ~4500 m) and Linzhi (LZT; altitude of ~3000 m) and Yorkshire pigs (5 months old, 75–90 kg) were also raised in Shannan (SNY) and Linzhi (LZY). The pigs were slaughtered according to commercial slaughter procedures by Woye Tibetan Pigs Co., Ltd. (Linzhi, China). The livers used for lipidomic analysis were purchased from Woye, ten livers of each breed and location were used (*n* = 40 in total). The edges of each liver (approximately 10 g) were clipped and immediately frozen in liquid nitrogen.

### 3.3. Lipid Extraction

Liver samples were removed from the liquid nitrogen and ground to a powder in a ceramic mortar using a grinding rod with liquid nitrogen. The sample powder (50 mg) was placed in a 2 mL centrifuge tube, to which 1 mL of lipid extract solution (methyl tert-butyl ether:methanol = 3:1, containing 10 μL of 5 μM internal lipid standard) was added. The sample solution was thoroughly ground by a homogenizer (MM400, Retsch, Haan, Germany) and then vortex-mixed (VORTEX-5, Kylin-Bell Lab Instruments Co., Ltd., Haimen, China) for 2 min. After adding 200 μL of water, the sample solution was vortexed again for 1 min and then centrifuged (5424R, Eppendorf, Hamburg, Germany) at 12,000× *g* (10 min at 4 °C). The supernatant (300 μL) was aspirated in a new 1.5-mL tube and concentrated to constant weight using a vacuum concentrator (Labconco, Kansas City, MO, USA). Finally, the lipid extract was reconstituted with 200 μL of mobile phase B (acetonitrile:isopropanol = 10:90, *v/v*; containing 0.1% formic acid and 10 mmol/L ammonium formate) for UPLC analysis.

Liver lipid extracts from two pigs within the same group were randomly and equally pooled as one biological replicate. A total of five biological replicates were prepared for each group, and the new merged samples were labelled SNT1–SNT5, SNY1–SNY5, LZT1–LZT5, and LZY1–LZY5, respectively. An additional four quality control samples (Mix1–Mix4) were prepared by mixing all samples equally, which were used to evaluate the repeatability of the lipidomic analyses. The internal standards used in this study and lipid identification information for all samples are shown in the Supplementary Materials (Tables S1 and S2 respectively).

### 3.4. UPLC-MS/MS

The lipidomic analysis was carried out with a UPLC-electrospray ionization (ESI)-MS/MS system (ExionLC AD and QTRAP^®^ System). UPLC separation was performed on a Thermo Accucore C30 column (2.6 μm, 2.1 mm × 100 mm) with a flow rate of 0.35 mL/min, and the column oven was set at 45 °C. The injection volume was 2 μL, mobile phase A was an acetonitrile/water solution (60:40, *v/v*; containing 0.1% acetic acid and 10 mmol/L ammonium formate), and mobile phase B was an acetonitrile/isopropanol solution (10:90, *v/v*; containing 0.1% formic acid and 10 mmol/L ammonium formate). The elution sequence was: 20% B at 0 min, 30% B at 2 min, 60% B at 4 min, 85% B at 9 min, 90% B at 14 min, 95% B at 15.5, 17.3 min, and 20% at 17.5, 20 min. The effluent was connected to the ESI source (500 °C) and the voltage was 5500 V (positive mode) or −4500 V (negative mode). The ion source gas 1 was set to 45 psi, gas 2 was set to 55 psi, and the curtain gas was set to 35 psi. The collision-induced ionization parameters were set as medium, and each ion pair in QTRAP was scanned and identified by the optimised declustering potential and collision energy.

### 3.5. Characterisation and Quantification of Lipid Molecular Species

Data acquisition of all samples was performed using Analyst software (1.6.3, AB Sciex, Foster City, CA, USA). Characterisation of lipid molecular species was carried out with a self-built database, MWDB, including more than 3000 lipid molecules (Metware Biotechnology Co., Ltd., Wuhan, China), and lipid molecular species were comprehensively identified based on the retention time, daughter and parent ion pair information, and characteristics of the MS/MS spectrum. The CAS number, KEGG pathway, and other supporting annotation information were determined, thus improving the accuracy of the qualitative analysis of the substance (Table S2). Lipid molecular species were quantified using the multiple reaction monitoring mode of triple-quadrupole mass spectrometry with the following parameters: <200 MS/MS transitions in each detection window; 3–50 ms dwell time of each MS/MS transition, depending on the compound’s retention time; and >10 collectable data points across a chromatographic peak.

### 3.6. Statistical Analysis

Multivariate statistical analyses, including PCA and OPLS-DA, were used to distinguish differences in the porcine liver lipidome [40]. A one-way analysis of variance was used for the comparison of lipid species among groups in GraphPad Prism 8.0 [41,42]. DALs between the groups were screened according to a fold change ≤0.5 or ≥2 and variable importance in projection >1. The KEGG database was used for the functional annotation and enrichment analysis of the DALs.

## 4. Conclusions

In this study, significant differences in liver lipids were found between Tibetan and Yorkshire pigs raised in different altitudinal environments. Among them, Tibetan pigs raised at a high altitude were found to be less efficient in lipid conversion, but also to actively produce energy to adapt to the cold plateau environment by performing more efficient FA oxidation. Conversely, Yorkshire pigs raised at a high altitude not only undergo more fat deposition but also exhibit spontaneous lipid oxidation to maintain body temperature. The environmentally adapted regulation of lipid metabolism in the livers of Tibetan and Yorkshire pigs has potential implications for their dietary nutritional value and meat quality attributes, which to some extent reflects the long-term selection and harmonious evolution of human dietary nutritional requirements and food-source species in different environments. The results of this study provide a theoretical basis for further understanding the adaptive regulatory mechanisms of Tibetan and Yorkshire pigs in response to different environments and also provide data to support the quality development of the highland livestock industry.

## Data Availability

Not applicable.

## Supplementary Materials

The following supporting information can be downloaded at: https://www.mdpi.com/article/10.3390/molecules28072991/s1, Figure S1: Total ion current (TIC) plots for mass spectrometric analysis of the same mass of quality control (QC) samples in positive (A) and negative ion modes (B). Figure S2: Pearson correlation analysis of QC samples. Figure S3: Principal component analysis (PCA) (A–D) and orthogonal partial least-squares discrimination analysis (OPLS-DA); (E–H) score plots of the liver lipidomes of different pig breeds raised at different altitudes. Table S1: The internal standards used in this study. Table S2: Lipid identification information for all samples.
