# Supplementary material for: Divergence of Liver Lipidomes in Tibetan and Yorkshire Pigs Living at Different Altitudes
Source: Molecules. 2023 Mar 27;28(7):2991. doi: 10.3390/molecules28072991 (PMC10095695; doi:10.3390/molecules28072991)
Supplement: Supplementary file 1 [file molecules-28-02991-s001.zip › molecules-2295973-supplementary.pdf]

Supplementary Materials

Divergence of Liver Lipidomes in Tibetan and Yorkshire Pigs Living at Different Altitudes

A

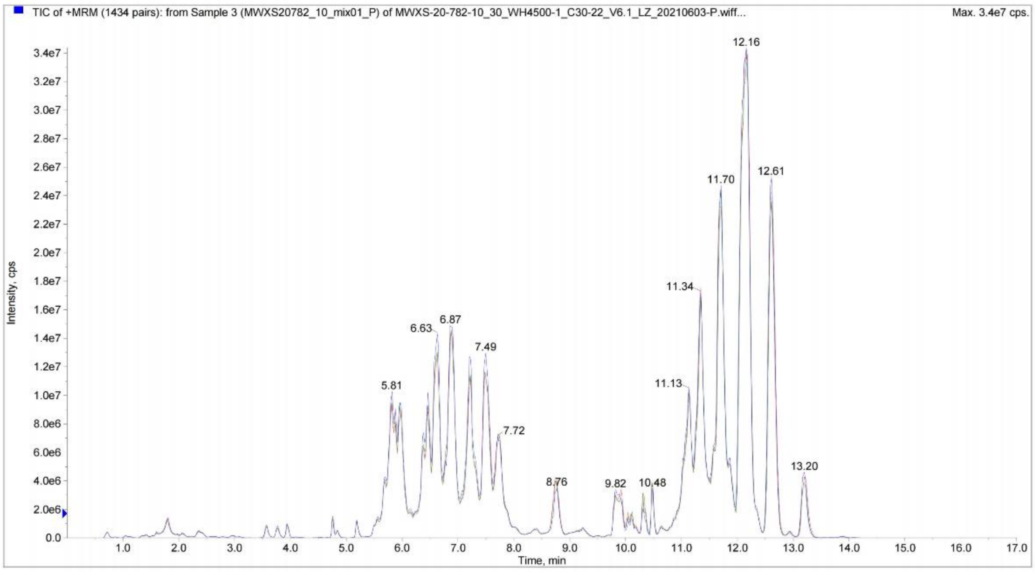

B

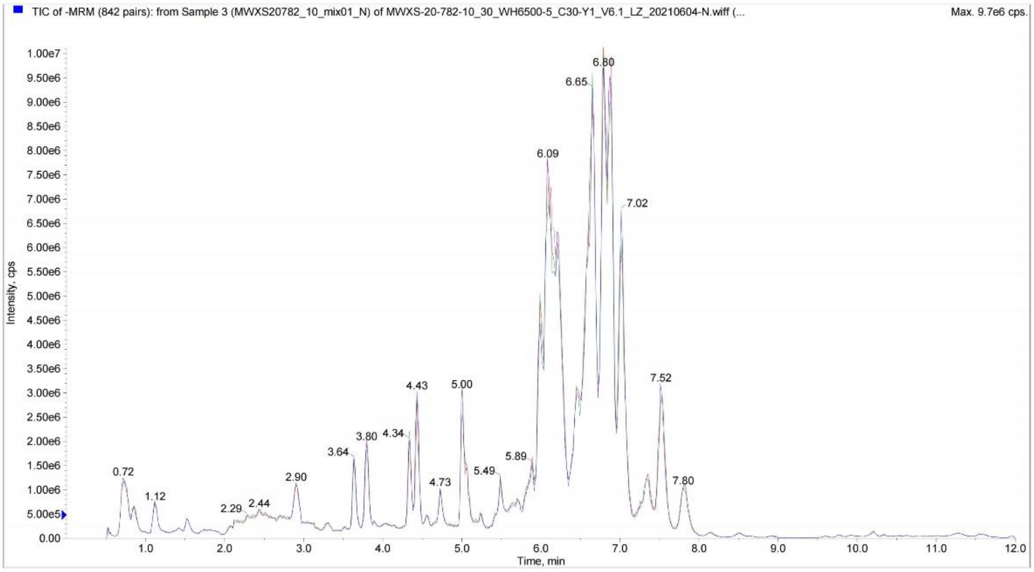

Figure S1: Total ion current (TIC) plots for mass spectrometric analysis of the same mass of quality control (QC) samples in positive (A) and negative ion modes (B).

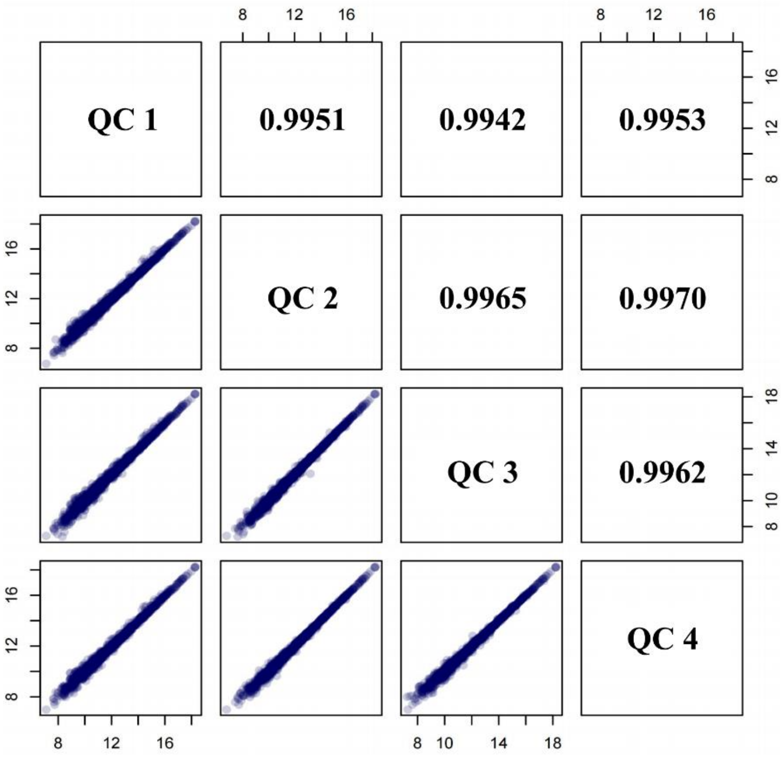

Figure S2: Pearson correlation analysis of QC samples.

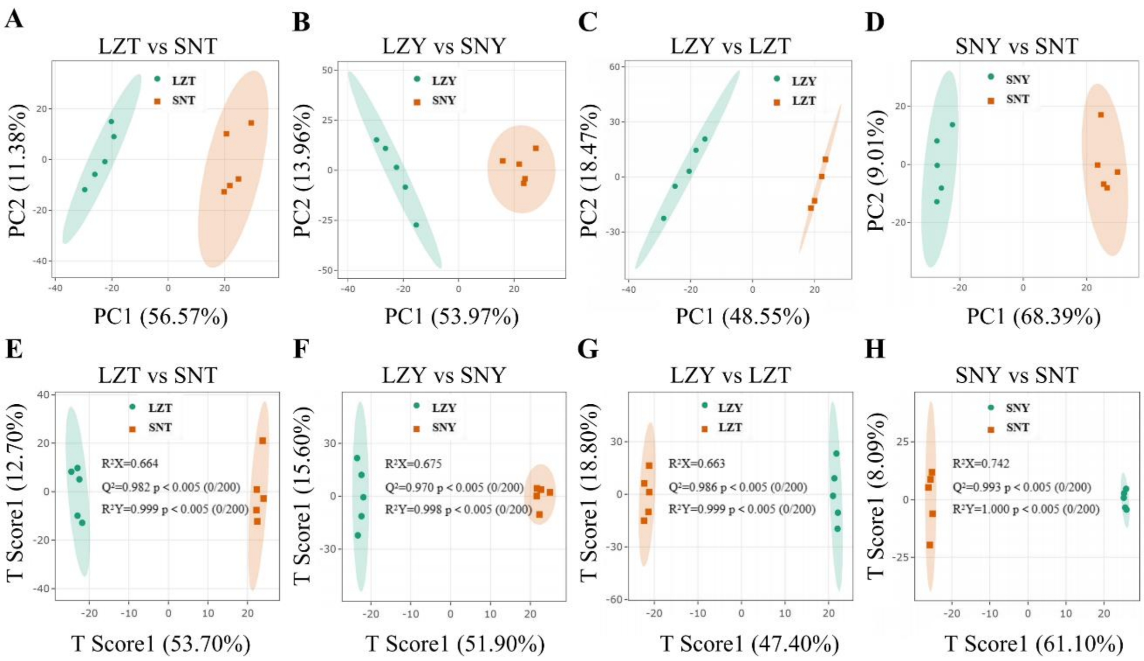

Figure S3. Principal component analysis (PCA) (A–D) and orthogonal partial least-squares discrimination analysis (OPLS-DA) (E–H) score plots of the liver lipidomes of different pig breeds raised at different altitudes. LZT, Linzhi (low-altitude, 3000 m) Tibetan pig; SNT, Shannan (high-altitude, 4500 m) Tibetan pig; LZY, Linzhi Yorkshire pig; SNY, Shannan Yorkshire pig, R^2X and R^2Y in the figure indicate the explanatory rates of the proposed model for the X and Y matrices, respectively, and Q^2 indicates the predictive power of the model, the corresponding *p*-values in parentheses are the frequencies of occurrence of random grouping models that outperformed the predictive power and explanatory rate of the present OPLS-DA model for the Y matrix, respectively, in 200 random permutation experiments.

Table S1: The internal standards used in this study – provided as a separate document.

Table S2: Lipid identification information for all samples – provided as a separate document.
